# The Influence of Food Texture and Liquid Consistency Modification on Swallowing Physiology and Function: A Systematic Review

**DOI:** 10.1007/s00455-014-9578-x

**Published:** 2014-10-25

**Authors:** Catriona M. Steele, Woroud Abdulrahman Alsanei, Sona Ayanikalath, Carly E. A. Barbon, Jianshe Chen, Julie A. Y. Cichero, Kim Coutts, Roberto O. Dantas, Janice Duivestein, Lidia Giosa, Ben Hanson, Peter Lam, Caroline Lecko, Chelsea Leigh, Ahmed Nagy, Ashwini M. Namasivayam, Weslania V. Nascimento, Inge Odendaal, Christina H. Smith, Helen Wang

**Affiliations:** 1 International Dysphagia Diet Standardisation Initiative (IDDSI) Working Committee, Brisbane, Australia; 2 Toronto Rehabilitation Institute – University Health Network, 550 University Avenue, #12-101, Toronto, ON M5G 2A2 Canada; 3 Department of Speech-Language Pathology, University of Toronto, Toronto, Canada; 4 School of Food Science and Nutrition, University of Leeds, Leeds, UK; 5 Sheikh Khalifa Medical City, Abu Dhabi, United Arab Emirates; 6 Zhejiang Gongshang University, Hangzhou, China; 7 School of Pharmacy, The University of Queensland, Brisbane, Australia; 8 Helen Joseph Hospital, Johannesburg, South Africa; 9 Medical School of Ribeirao Preto - University of Sao Paulo, Ribeirao Preto, Brazil; 10 Sunny Hill Health Centre, Vancouver, BC Canada; 11 Department of Mechanical Engineering, University College London, London, UK; 12 Peter Lam Consulting, Vancouver, BC Canada; 13 Faculty of Land and Food Systems, University of British Columbia, Vancouver, BC Canada; 14 National Health Service Commissioning Board Special Health Authority, London, UK; 15 Fayoum University, Fayoum, Egypt; 16 University of KwaZulu-Natal, Durban, South Africa; 17 Division of Psychology and Language Sciences, University College London, London, UK

**Keywords:** Deglutition, Deglutition disorders, Dysphagia, Texture modification, Systematic review

## Abstract

Texture modification has become one of the most common forms of intervention for dysphagia, and is widely considered important for promoting safe and efficient swallowing. However, to date, there is no single convention with respect to the terminology used to describe levels of liquid thickening or food texture modification for clinical use. As a first step toward building a common taxonomy, a systematic review was undertaken to identify empirical evidence describing the impact of liquid consistency and food texture on swallowing behavior. A multi-engine search yielded 10,147 non-duplicate articles, which were screened for relevance. A team of ten international researchers collaborated to conduct full-text reviews for 488 of these articles, which met the study inclusion criteria. Of these, 36 articles were found to contain specific information comparing oral processing or swallowing behaviors for at least two liquid consistencies or food textures. Qualitative synthesis revealed two key trends with respect to the impact of thickening liquids on swallowing: thicker liquids reduce the risk of penetration–aspiration, but also increase the risk of post-swallow residue in the pharynx. The literature was insufficient to support the delineation of specific viscosity boundaries or other quantifiable material properties related to these clinical outcomes. With respect to food texture, the literature pointed to properties of hardness, cohesiveness, and slipperiness as being relevant both for physiological behaviors and bolus flow patterns. The literature suggests a need to classify food and fluid behavior in the context of the physiological processes involved in oral transport and flow initiation.

## Introduction

The use of texture-modified foods and thickened liquids has become a cornerstone of clinical practice to address dysphagia (swallowing impairment) [1, 2]. The principle behind this pervasive practice arises from the assumption that modifying the properties of normal foods and liquids will make them easier and safer to swallow. In the case of liquids, it is widely accepted that thin liquids (such as water) pose safety challenges for people with dysphagia because they flow quickly [3, 4]. The speed of bolus flow from the mouth into the pharynx may be sufficiently fast that it does not provide enough time for the person to engage airway closure before the bolus arrives at the entrance to the larynx and airway. Thickened liquids are frequently recommended with the goal of slowing down the flow of liquids to allow more time for airway closure [4, 5]. Conversely, very thick liquids and solid food materials may require greater strength in terms of the tongue propulsive forces that are used to drive material through the oropharynx. If a person has reduced tongue strength or reduced pharyngeal muscle strength, this is felt to constitute a risk for residues to remain behind in the recesses of the pharynx after a swallow [4, 6–8]. Similarly, solid foods that require chewing may prove challenging for people with dental issues or weakness in the masticatory muscles. Alteration of the properties of solid foods (by dicing, chopping, mincing or pureeing) is a common approach to making these materials easier for oral processing and swallowing.

The widespread use of texture modification as a clinical intervention has created a need to establish clear terminology to describe the target consistencies that are recommended for patients with dysphagia. In the absence of clear terminology and definitions to guide both the production/preparation and the clinical use of modified food textures and liquid consistencies, several countries have developed taxonomies or classification systems, disseminated in the form of clinical guidelines [9–14]. However, different countries have developed different systems of classification [15]. Recognition of the need to agree on terminology both within and across geographic jurisdictions has led to the establishment of the International Dysphagia Diet Standardisation Initiative (www.iddsi.org). The IDDSI task force has set a goal of developing global standardized terminology and definitions for texture-modified foods and thickened liquids for individuals with dysphagia of all ages, in all care settings, and all cultures.

The majority of existing guidelines for texture terminology have been developed based on input derived from expert opinion, focus groups, and interviews with clinicians [10, 12–14]. In addition to best practice and expert or consensus opinion, some guidelines have drawn on evidence from the literature to support their nomenclature [11]. However, it has been seven years since the last review of evidence from the literature [11]. In addition to consensus opinion, the IDDSI project has a goal to consider current empirical evidence when determining the number and characteristics of the terms that should be used in a recommended taxonomy of thickened liquids and texture-modified foods for clinical use. This article describes a systematic review of the literature that has been conducted to identify high quality scientific evidence regarding the influence of bolus consistency on swallowing function and/or physiology, either in healthy or impaired participants. For the purposes of this review, the term *swallowing function* is used to refer either to swallowing safety (i.e., swallowing without material being aspirated into the airway) and/or swallowing efficiency (i.e., swallowing material in a reasonable timeframe without leaving residual behind in the mouth or pharynx). The term *swallowing physiology* is used to refer to the biomechanical components of swallowing behavior, such as hyoid and laryngeal movement, tongue function or upper esophageal sphincter opening, which ultimately contribute to functional swallowing outcomes. With respect to labeling levels or categories of texture-modified liquids in this article, we will use the labels “thin”, “nectar-thick”, “honey-thick”, “pudding-thick/puree/paste”, “soft solids” and “hard solids” because these were the terms encountered most frequently in the research literature. It is acknowledged that terms like these are not culturally neutral or transparent, and are open to different interpretations. A previous publication by the IDDSI task force provides tables comparing terms across different guidelines and geographical jurisdictions [15]. For the purposes of this review, the term “nectar-thick” should be interpreted to refer to an initial degree of thickening (i.e., slightly thicker than thin or unthickened liquids), while the terms “honey-thick” and “pudding-thick” refer to progressively greater degrees of thickening, respectively.

The purpose of this review was to identify and review articles describing eating and swallowing in humans of any age, in which at least two different consistencies of food and/or liquid had been tested, and in which objective measures of swallowing function or physiology were reported for different bolus consistencies. The review also included articles describing the rheological or material characteristics of food or liquid stimuli after oral processing (i.e., at the point of swallowing). Articles describing the measures of interest either in healthy people, and/or in people with oropharyngeal dysphagia, without any restrictions related to diagnostic etiology were included. There were no restrictions imposed on the diagnostic or instrumental methods used, provided that some form of objective measurement was performed to capture the parameters of interest. Articles in all languages were accepted, based on the fact that the IDDSI working committee had the necessary expertise to provide or access translation for many non-English languages. Once identified, the intent was to evaluate the selected articles to determine evidence-informed answers to the following research questions:

1. Is there evidence to support or refute a functional or behavioral change resulting from the thickening of liquids and/or texture modification of foods? If yes, how many and which levels of thickening or texture modification are supported by evidence, and what is the quality of evidence)?
2. Does the literature provide trustworthy objective measures (e.g., viscosity, density, yield stress, texture analysis, or other physical measures) to guide the definition of different levels of thickened liquids and texture modified foods?
3. Does the available evidence have application across the lifespan, or is it specific to particular subpopulations, defined either by age or diagnosis?
4. What are the gaps in the literature regarding thickening of liquids and/or texture modification of food as a strategy to manage dysphagia?

## Methods

A comprehensive literature search for literature published between 1985 and January, 2013 was conducted using multiple search engines, including Ovid MEDLINE(R), Ovid MEDLINE(R) In-process and other non-indexed citations, AMED (Allied and Complementary Medicine), EMBASE, Health and Psychosocial Instruments, and PsycINFO. The search was also conducted in Scopus using the following subject area limits: medicine, agricultural and biological sciences, pharmacology etc., chemistry, nursing, neuroscience, chemical engineering, engineering, health professions, psychology, materials science, multidisciplinary, dentistry. Search terms were broadly specified with the goal of finding as much relevant literature as possible, and included MeSH Headings of “Swallowing” or “Deglutition” or “Dysphagia”. Inclusion of one or more key-word (Scopus) or text terms (all other search engines) was also specified with the goal of focusing the results on the topic of interest. These terms were: “Visco*”; “Bolus”; “rheo*”; “dens*”; “yield*”; “fluid*”; “mechani*”; “elastic*”; “Newton*”; “carbohydrate”; “colloid*”; “starch”; “gum”; “alginate”; “cohes*”; “thick*”; “consisten*”; “nectar”; “honey”; “puree*”; “pudding”; “thin”; “spoon”; “liqui*”; “textur*”; “smooth*”; “mince*”; “soft”; “dice*”; “chop*”; “fibr*”; “fibe*”; “bread” or “solid*”. The asterisk used at the end of each search term stem allows for different word endings; for example, the stem “textur*” searches for the words “texture”, “textured” or “textural”. Terms were nominated by the authors based on their professional experience and following consultation with peers. The final set of search terms was intended to capture concepts and terms known to be used in the food oral processing and dysphagia research communities to describe food or liquid properties. It should be noted that terms related to choking, airway obstruction, or asphyxiation were not included in the search strategy for this review.

**Fig. 1:**
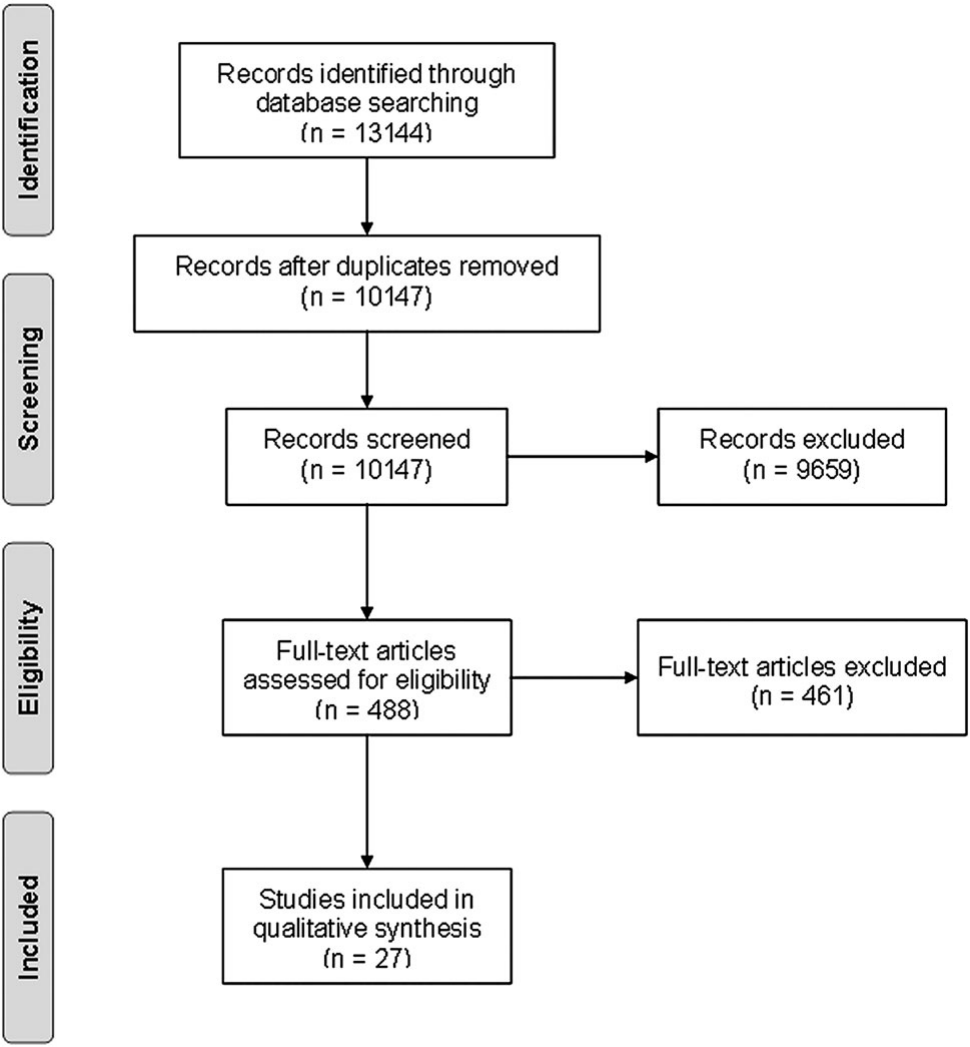
PRISMA flow diagram of the search process used in this systematic review

As a step in measuring construct validity, and to confirm that the search was succeeding in finding important articles from the dysphagia and food processing research literature, members of the IDDSI working committee generated a list of known articles that they expected should have been found in the course of the search. A cross-check of these nominated articles with the search results revealed gaps in the search results with respect to articles describing swallowing in children or arising from the food oral processing literature. Consequently, two additional searches were conducted using the same search engines. The first of these sought articles under the MeSH search term “eating and feeding disorders of childhood” while the second search specified the additional MeSH term of “food texture” in combination with the original search terms. Figure 1 summarizes the yield of this literature search strategy according to the criteria laid out in the 2009 PRISMA guideline for systematic reviews [16].

This set of 10,147 non-duplicate articles was subjected to an initial screening review to identify a sub-set of articles for full-text review. A team of three research assistants (LG, CL and HW) screened the titles and abstracts of the complete search yield of 10,147 non-duplicate articles to determine relevance, defined as an article describing a measurement of human swallowing using more than one consistency of food or liquid. This initial screening was conducted blindly in duplicate. Articles were included if they were identified as relevant by at least one reviewer. This led to a set of 488 articles selected for more detailed full-text review. These 488 articles were assigned to an international team of 10 raters who each reviewed between 40 and 70 articles for relevance and quality using a questionnaire administered using SurveyMonkey^®^ (see Table 1). Training in completion of the relevance and quality ratings was provided via teleconference with subsequent email support from the lead author (CMS). The questions addressed during the full-text review are listed in Table 1 and led to a final subset of 36 articles selected for qualitative synthesis. The qualitative synthesis included extraction of trends from the data both within and across specific participant subgroups (e.g., healthy, stroke patients) and critical appraisal of the risk of bias at both the study level and the outcome level based on the food and liquid consistencies and the measurement instruments used in each study. Due to the wide variety of instrumental methods used to measure swallowing behaviors, and the wide variety of foods and liquids used in the selected studies, it was not possible to undertake a quantitative analysis of results across studies. As the final step in this systematic review, the interpretations arising from the qualitative synthesis were shared with members of the IDDSI international working committee for reaction and discussion.

**Table 1:** Questions addressed during the full-text relevancy and quality review

Number	Question	Clarifying instructions
1	Is the article is a peer-reviewed manuscript in a journal?^a^	Conference abstracts should be excluded
2	Does the article report at least one empirical measure of swallowing behavior in humans for at least two textures or consistencies?^a^	Studies reporting a single item, e.g., ONLY water swallows, ONLY thin liquid barium swallows, or ONLY saliva swallows should be excluded. Reviews without original data should be excluded
3	What were the different stimuli tested?	
4	Does the article describe the stimuli that were used in a way that can be replicated, or provide specific quantitative measures of food/liquid texture characteristics (such as viscosity)?^b^	A descriptive label such as “nectar thick” is not adequate unless the brand name of a commercial product is provided, actual rheological measurements of the product are reported, or a replicable recipe is reported
5	What was the research question?	Please state as clearly as possible
6	Are participant eligibility criteria clearly specified? (age, sex, etiology, etc.)^b^	
7	Are the participant groups clearly delineated and described?^b^	
8	Are other relevant characteristics of the food and fluid stimuli reported? (temperature, taste, volume/bite size, administration method, aroma)^b^	
9	Were all conditions and measurements applied similarly to all participants?^a^	
10	Was the order of stimulus presentation randomized?^b^	
11	Are precise and repeatable methods of measuring swallowing operationally defined?^a^	Examples: tongue pressures were measured using the iowa oral performance instrument; ultrasound measures of tongue height were measured
12	Does the manuscript report data for at least one parameter for the majority of the participants enrolled (80 % or more of the participants)?^a^	This question is asking whether the data are complete or whether there are a lot of missing data
13	Was there sufficient data collected for each condition?^a^	More than 1 swallow of each condition is required at a minimum
14	Are both point measures (e.g., mean, median) and measures of variability (e.g., standard deviation or confidence interval) reported for at least one key outcome variable?^b^	Please summarize the types of statistics that were reported
15	What is the overall conclusion or main finding of this study related to swallowing or oral processing and food/fluid texture?	

^a^Questions indicating properties that require a ‘‘Yes’’ response for inclusion in the qualitative synthesis

^b^Questions capturing quality parameters related to the review

## Results

### Participant Characteristics

Demographic information regarding the participants of the 36 studies selected for qualitative synthesis is shown in Table 2. Notably, only three of these studies described swallowing or oral processing in children; one of these was a study of swallowing in premature infants [17], while a second [18] explored differences in chewing behaviors in infants aged 6 months to 2 years of age. The third study involving children explored oral processing behaviors in two groups of typically developing girls aged 5 and 8 years old, as well as a control group of healthy adult women [19]. Of the 29 studies describing swallowing or oral processing in adults, 27 reported data for healthy adult participants [19–45], with two studies restricting their focus to denture wearers [46, 47]. A total of 10 studies reported data for adults with dysphagia [20–24, 48–52]. Four of these studies described swallowing in stroke patients in comparison to healthy controls [21–24] and a 5th paper described a group of patients with dysphagia secondary to Chagas’ disease, again with comparison to a group of healthy controls [20]. Two papers reported data for individuals with dysphagia related to head and neck cancer, in one case following surgical resection of the soft palate [48] and the second exploring post-radiation dysphagia in patients treated for nasopharyngeal carcinoma [49]. The remaining papers described swallowing in patients with Parkinson’s disease [50], in unspecified neurogenic dysphagia [51], or in unspecified dysphagia [52]. Sample sizes ranged from 3 [25] to 205 [24] participants.

**Table 2:** Demographics of the study samples of the 36 articles selected for detailed review

Author	Year	Title	Sample size	Healthy children?	Healthy adults?	Pediatric patient sample?	Adult patient sample?
Goldfield et al. [17]	2013	Preterm infant swallowing of thin and nectar-thick liquids: changes in lingual-palatal coordination and relation to bolus transit	10			Premature infants	
Gisel [18]	1991	Effect of food texture on the development of chewing of children between 6 months and 2 years of age	143	✓			
Ruark et al. [19]	2002	Bolus consistency and swallowing in children and adults	30	✓	✓		
Lee et al. [52]	2012	Is swallowing of all mixed consistencies dangerous for penetration–aspiration?	29				Adults with dysphagia (no etiology specified)
dos Santos et al. [20]	2011	Videofluoroscopic evaluation of swallowing in Chagas’ disease	32		✓		Chagas’ disease
Barata et al. 48]	2013	Swallowing, speech and quality of life in patients undergoing resection of soft palate	23				Head and neck cancer patients post soft palate resection and reconstruction
Lin et al. [49]	2011	Effects of functional electrical stimulation on dysphagia caused by radiation therapy in patients with nasopharyngeal carcinoma	20				Nasopharyngeal carcinoma patients post radiation
Chen et al. [51]	1992	Clinical and videofluoroscopic evaluation of swallowing in 41 patients with neurologic disease	41				Neurogenic disorders
Troche et al. [50]	2008	Effects of bolus consistency on timing and safety of swallow in patients with Parkinson’s disease	10				Parkinson’s disease
Kim and Han [21]	2005	Influence of mastication and salivation on swallowing in stroke patients	20		✓		Stroke patients
Bisch et al. [22]	1994	Pharyngeal effects of bolus volume, viscosity and temperature in patients with dysphagia resulting from neurological impairment and in normal subjects	28		✓		Stroke patients
Oommen et al. [23]	2011	Stage transition and laryngeal closure in poststroke patients with dysphagia	72		✓		Stroke patients
Bingjie et al. [24]	2010	Quantitative videofluoroscopic analysis of penetration–aspiration in post-stroke patients	205		✓		Stroke patients
Karkazis and Kossioni [47]	1998	Surface EMG activity of the masseter muscle in denture wearers during chewing of hard and soft food	9		Denture wearers		
Karkazis [46]	2002	EMG activity of the masseter muscle in implant supported overdenture wearers during chewing of hard and soft food	6		Edentulous participants with mandibular overdentures supported by implants		
Anderson et al. [39]	2002	The effects of bolus hardness on masticatory kinematics	26		✓		
Nagatomi et al. [40]	2008	Multivariate analysis of the mechanical properties of boluses during mastication with the normal dentitions.	12		✓		
Karkazis and Kossioni [41]	1997	Re-examining of the surface EMG activity of the masseter muscle in young adults during chewing of two test foods.	22		✓		
Hoebler et al. [42]	1998	Physical and chemical transformations of cereal food during oral digestion in human subjects.	12		✓		
Funami et al. [43]	2012	Texture design for products using food hydrocolloids	9; 7		✓		
Ashida et al. [44]	2007	Analysis of physiological parameters of masseter muscle activity during chewing of agars in healthy young males.	18		✓		
Linden et al. [25]	1989	Bolus position at swallow onset in normal adults: preliminary observations	3		✓		
Reimers-Neils et al. [26]	1994	Viscosity effects on EMG activity in normal swallow	5		✓		
Taniwaki et al. [27]	2013	Acoustic analysis of the swallowing sounds of food with different physical properties using the cervical auscultation method	6		✓		
Saitoh et al. [45]	2007	Chewing and food consistency: Effects on bolus transport and swallow initiation.	15		✓		
Steele and Van Lieshout [28]	2004	Influence of bolus consistency on lingual behaviors in sequential swallowing	8		✓		
Steele and Van Lieshout [29]	2005	Does barium influence tongue behaviors during swallowing?	8		✓		
Igarashi et al. [30]	2010	Sensory and motor responses of normal young adults during swallowing of foods with different properties and volumes	12		✓		
Ishida et al. [31]	2002	Hyoid motion during swallowing: factors affecting forward and upward displacement	12		✓		
Lee et al. [32]	2010	Effects of liquid stimuli on dual-axis swallowing accelerometry signals in a healthy population	17		✓		
Butler et al. [33]	2004	Effects of viscosity, taste, and bolus on swallowing apnea duration of normal adults	22		✓		
Chi-Fishman and Sonies [53]	2002	Effects of systematic bolus viscosity and volume changes on hyoid movement kinematics	31		✓		
Youmans et al. [35]	2009	Differences in tongue strength across age and gender: is there a diminished strength reserve?	96		✓		
Inagaki et al. [36]	2008	Influence of food properties and body posture on durations of swallowing-related muscle activities	9		✓		
Inagaki et al. [37]	2009a^a^	Activity pattern of swallowing-related muscles, food properties and body position in normal humans	9		✓		
Inagaki et al. [38]	2009b^a^	Influence of food properties and body posture on swallowing-related muscle activity amplitude	9		✓		

^a^The three studies by Inagaki et al. appear to deal with data from a single experiment

### Stimulus Characteristics

The various food and liquid stimuli used in the studies selected for inclusion in the qualitative synthesis are summarized in Tables 3 (radio-opaque liquid stimuli), 4 (non-opaque liquid stimuli), and 5 (solid stimuli). Of the 36 studies selected for detailed review, seven reported comparative data for swallows of thin liquid (either barium, water or juice) and an extremely thick liquid (i.e., pureed or spoon-thick consistency) [19, 26, 33, 35, 48, 51, 53]. A total of 13 articles described swallowing measures for a narrower contrast, i.e., thin liquid compared to either a mildly thick liquid (also known as nectar-thick) [17, 19, 23, 26, 28–30, 32, 35, 48, 51, 53] and/or a moderately thick liquid (also known as honey-thick) [28–30, 32, 33, 35, 53], with six of these articles including both mildly thick and moderately thick liquids [28–30, 32, 35, 53]. In terms of solid stimuli, which were explored in a total of 18 studies (Table 5) [18, 19, 21, 24, 27, 31, 39–48, 51, 52], there were effectively no stimuli that were the same in any two or more studies. Solid foods ranged from items that were described by authors as being softer (i.e., banana with barium paste [31]; cooked rice mixed with barium [52]; corned beef [45]; gummy bears [19]; konjac jelly [27], or gelatin cubes [18]) to items at the harder end of the continuum (e.g., fresh raw carrots [27]; biscuits or cookies [24, 31, 48, 51] or peanuts [31]). The description of certain items as “softer” in these studies illustrates the subjectivity with which texture descriptors may be applied. For example, “crisp, peeled apple” were described as being “softer” [46] in comparison to raw carrot [46], despite the fact that a crisp apple would not generally be regarded as a soft texture. Three studies, all originating from Asia, explored the combination of solid and liquid consistencies using either corned beef in a liquid barium [45], a thick rice gruel (the consistency of which was not further described) [21] or 12 g of cooked rice added to 100 ml of liquid barium [52].

**Table 3:** Radio-opaque liquid stimuli used in studies exploring swallowing of different consistencies

Author	Thin (opaque)	Nectar-thick (opaque)	Honey-thick (opaque)	Pudding-thick/paste/puree (opaque)
Bingjie et al. [24]	Thin liquid barium			Applesauce mixed with barium
Bisch et al. [22]	Liquid barium			Pudding-thick barium
Chen et al. [51]	1:1 dilution of E-Z-HD (120 w/v, E-Z-EM) “with the viscosity of water”	Polibar (100 % w/v, E-Z-EM) “with a viscosity similar to that of syrup”		Esophotrast barium paste (100 % w/v) “with the consistency of pudding”
dos Santos et al. [20]	Bariogel 100 % liquid barium sulfate			30 ml of 100 % liquid barium sulfate with 3 g of Nutilis thickener (Nutricia)
Goldfield et al. [17]	Barium sulfate diluted in a 50 % ratio with 5 % glucose in water to simulate human milk or formula	Liquid E-Z Paque, EZ-E-M		
Ishida et al. [31]	E-Z-HD barium suspension diluted to 50 % w/v ratio			8 g of chicken spread (Underwood Chunky Chicken) mixed with a little Esophotrast barium paste
Kim and Han [21]	Liquid barium (barium sulfate, 140 g/100 ml) mixed equally with water			
Lee et al. [52]	5 ml of l 140 g/100 ml liquid barium sulfate (Raydix) plus 100 ml of normal saline			
Lee et al. [32]	40 % w/v thin liquid barium suspension (prepared using water and Liquid Polibar™ barium, E-Z-EM)	Commercially pre-thickened nectar-thick apple juice (RESOURCE^®^, Novartis Nutrition)		
Lin et al. [49]	Thin barium: 340 g E-Z-HD powder (E-Z-EM, Inc.) with 65 ml of water			Paste barium: 15 ml of the thin barium preparation plus an additional 12 ml of E-Z-HD powder
Linden et al. [25]	Barosperse 50 % “with a viscosity similar to water”			Esophotrast
Oommen et al. [23]	A mixture of water and E-Z-HD barium sulfate powder with a viscosity of 14 mPa s	A mixture of thickened juice and E-Z-HD barium sulfate powder with a viscosity of 187 mPa s		
Saitoh et al. [45]	Liquid barium (not specified)			
Steele and Van Lieshout [29]	EZ-HD 8 % w/w, 250 % w/v barium suspension (density: 2.54 g/cc; yield stress: 0.338 Pa, viscosity: 351 mPa s @ 25/s)	Novartis RESOURCE^®^ Nectar-thick Apple juice mixed four parts to one with EZ-H–D 250 % w/v barium sulfate powder (E-Z-EM Therapex): (density: 1.15 g/cc; yield stress: 1.055 Pa; viscosity: 863 mPa s @ 25/s	Novartis RESOURCE^®^ Honey-thick Apple juice mixed four parts to one with EZ-H–D 250 % w/v barium sulfate powder (E-Z-EM Therapex): (density: 1.13 g/cc; yield stress: 2.109 Pa; viscosity: 1541 mPa s @ 25/s	
Troche et al. [50]	(Liquid E-Z Paque Barium Sulfate Suspension; 60 % w/v, 41 % w/w (E-Z-EM)			(Varibar Pudding-barium Sulfate Esophageal Paste, 230 ml 40 % w/v, 30 % w/w (E-Z-EM)

**Table 4:** Liquid stimuli used in non-radiographic studies of swallowing of different consistencies

Author	Thin	Nectar-thick	Honey-thick	Pudding-thick/paste/puree
Barata et al. [48]	Juice	Nectar-thick liquid		Puree
Butler et al. [33]	Apple juice (1 mPa s)		Honey-thick thick apple juice (Diamond Crystal Medical Food): viscosity of 1,100–1,900 mPa s	Applesauce (Lucky Leaf, Peach Glen, PA)
Chi-Fishman and Sonies [53]	Lemon-flavored water, 7 mPa s	Lemon-flavored water thickened with corn-starch to 243–260 mPa s	Lemon-flavored water thickened with corn-starch to 724–759 mPa s	Lemon-flavored water thickened with corn-starch to spoon-thick, 2760–2819 mPa s
Gisel [18]				Unsweetened applesauce
Igarashi et al. [30]	A test food consisting of water, 0.15 % citric acid, 9 % sucrose, 0.04 % flavor	A test food consisting of water, 0.17 % citric acid, 13.5 % sucrose, 0.08 % flavor and 1.5 % of a thickening agent comprised of guar gum, tara gum, carrageenan, xanthan gum, starch and dextrin	A test food consisting of water, 0.24 % citric acid, 15 % sucrose, 0.12 % flavor and 3 % of a thickening agent comprised of guar gum, tara gum, carrageenan, xanthan gum, starch and dextrin	
Inagaki et al. [36]		2 % concentration of mousse-up thickening agent in 100 ml distilled water	6 g of mousse-up thickening agent dissolved in 100 ml distilled water (5.7 %)	10 g of mousse-up thickening agent dissolved in 100 ml distilled water (9.1 %)
Inagaki et al. [37]		2 % concentration of mousse-up thickening agent in 100 ml distilled water	6 g of mousse-up thickening agent dissolved in 100 ml distilled water (5.7 %)	10 g of mousse-up thickening agent dissolved in 100 ml distilled water (9.1 %)
Inagaki et al. [38]		2 % concentration of mousse-up thickening agent in 100 ml distilled water	6 g of mousse-up thickening agent dissolved in 100 ml distilled water (5.7 %)	10 g of mousse-up thickening agent dissolved in 100 ml distilled water (9.1 %)
Kim and Han [21]				(1) Pudding; (2) curd-type yogurt
Lee et al. [32]	Water		Commercially pre-thickened honey-thick apple juice (RESOURCE^®^, Novartis Nutrition)	
Reimers-Neils et al. [26]	Fruit juice (Kraft)	Tomato juice (Libby’s)		1) Apple sauce (The Jewel Companies); 2) Chocolate pudding (Beatrice/Hunt-Wesson Inc.); 3) Cheese spread (Nabisco); 4) Creamy peanut butter (Best Foods, CPC International Inc.)
Ruark et al. [19]	Water	One-part apple juice to one-part applesauce (The Kroger Company)		Cheese spread (Easy Cheese, Nabisco Foods)
Steele and Van Lieshout [28]	(1) Water (density: 0.993 g/cc; yield stress: 0.000 Pa; viscosity: 12 mPa.s @ 45/s); (2) Apple juice (density: 1.007 g/cc; yield stress: 0.029 Pa; viscosity: 5 mPa s @ 45/s)	(1) Sealtest* 1 % M.F. Chocolate Milk (density: 1.053 g/cc; yield stress: 0.052 Pa; viscosity: 324 mPa.s @ 45/s). (2) Novartis RESOURCE^®^ Nectar-thick Apple juice (density: 1.067 g/cc; yield stress: 0.264 Pa; viscosity: 325 mPa s @ 45/s)	(1) Novartis RESOURCE^®^ Original Honey-thick Dairy made with 2 % reduced fat milk (density: 1.04 g/cc; yield stress: 0.0 Pa; viscosity: 867 mPa s @ 45/s). (2) Novartis RESOURCE^®^ Honey-thick Apple juice (density: 1.073 g/cc; yield stress: 1.424 Pa; viscosity: 785 mPa s @ 45/s)	
Steele and Van Lieshout [29]	Water (density: 0.993 g/cc; yield stress: 0.000 Pa; viscosity: 12 mPa s @ 45/s)	Novartis RESOURCE^®^ Nectar-thick Apple juice (density: 1.067 g/cc; yield stress: 0.264 Pa; viscosity: 466 mPa s @ 25/s)	Novartis RESOURCE^®^ Honey-thick Apple juice (density: 1.073 g/cc; yield stress: 1.424 Pa; viscosity: 1140 mPa s @ 25/s)	
Taniwaki et al. [27]	Water			Yogurt (Bio presweetened, Danone Japan Co. Ltd., Tokyo, Japan) Viscosity: 3.2 Pa @ 0.0061/s
Youmans et al. [35]	Water	Novartis resource nectar-thick apple juice	Novartis resource honey-thick apple juice	Puree (“the consistency of applesauce”)

**Table 5:** Solid stimuli (both radio-opaque and non-opaque) used in studies of oral processing and swallowing

Author	Opaque/non-opaque	Mixed consistency	Soft solids	Hard solids
Anderson et al. [39]	Non-opaque		Soft chewing gum (hardness = 440 g measured on a durometer)	Hard chewing gum (hardness = 670 g measured on a durometer)
Ashida et al. [44]	Non-opaque		(1) Low concentration ordinary agar gel; (2) Low concentration mixture of agar, k-carrageenan, locust bean gum and glucose	(1) High concentration ordinary agar gel; (2) High concentration mixture of agar, k-carrageenan, locust bean gum and glucose
Barata et al. [48]	Non-opaque			Toasted biscuits
Bingjie et al. [24]	Opaque			Biscuits coated with barium
Chen et al. [51]	Opaque			Cookie (Lorna Done, Nabisco) coated with barium paste
Funami et al. [43]	Non-opaque		(1) Agar gel containing a mixture of gellan gum and psyllium seed gum: hardness level of 1,000 Pa at 67 % strain. (2) De-acylated gellan gum: hardness level of 1,000 Pa at 67 % strain	(1) Agar gel containing a mixture of gellan gum and psyllium seed gum: hardness level of 4,000 Pa at 67 % strain. (2) De-acylated gellan gum: hardness level of 4000 Pa at 67 % strain
Gisel [18]	Non-opaque		Gelatin cubes: consistency reported to “melt slowly in the mouth”	Cheerios cereal
Hoebler et al. [42]	Non-opaque		(1) White wheat bread; (2) durum wheat spaghetti	
Ishida et al. [31]	Opaque		8 g of banana with a light superficial coating of Esophotrast barium paste.	(1) Shortbread cookie (Walker’s Shortbread Ltd.) with a light coating of Esophotrast barium paste; (2) Unsalted dried peanuts mixed with Esophotrast barium paste
Karkazis [46]	Non-opaque			(1) Fresh raw carrots; (2) crisp peeled apples.
Karkazis and Kossioni [47]	Non-opaque		Crisp peeled apples in 1 cm^3^ pieces	Fresh raw carrots in 1 cm^3^ pieces
Karkazis and Kossioni [41]	Non-opaque		Non-adhesive chewing gum	Raw carrots
Kim and Han [21]	Non-opaque	Thick rice gruel		
Lee et al. [52]	Opaque	12 g cooked rice mixed with 100 ml liquid barium	24 g cooked rice mixed with 5 ml liquid barium	
Nagatomi et al. [40]	Non-opaque		Cheese	(1) Rice crackers; (2) Peanuts
Ruark et al. [19]	Non-opaque		Gummy bear (Favorite Brands International)	
Saitoh et al. [45]	Opaque	Corned beef mixed with liquid barium	Corned beef mixed with powder barium	
Taniwaki et al. [27]	Non-opaque		Konjac jelly (Konnyaku Batake, Mannan Life Co. Ltd., Gunma, Japan). Yield stress: 10 Pa @ 0.0061/s	

Given the available data, it appears reasonable to synthesize observations regarding differences in swallowing physiology and function across the spectrum of liquid consistencies, from the thin to the extremely thick end of the continuum. However, caution is warranted with respect to delineating quantitative values to capture levels or categories of liquids along this continuum, based on incomplete reporting and the variety of methods and measures used to characterize liquid flow in the studies reviewed. This variety challenges the idea that the stimulus labels used in the literature (e.g., thin, nectar-thick, honey-thick) map to defined ranges of flow. For example, a wide variety of different studies reported using thin liquid barium, but where recipes were reported, these used different concentrations of barium and different dilutions with water or other thin liquids. Insufficient information was provided in the majority of these studies to support recipe replication, or to calculate the weight to volume concentrations of the resulting barium suspensions. Furthermore, given that commercial barium preparations frequently involve additional components to reduce foaming or aid suspension, including gums and starches, viscosity cannot be presumed without additional information.

Very few studies provided objective measures of stimulus characteristics such as viscosity, yield stress, or density (see Tables 3, 4). In several cases, the authors used metaphors to describe the apparent viscosities of stimuli, such as “with a viscosity similar to water”, but failed to provide adequate evidence to support these descriptions. Indeed, several of the metaphors used were scientifically implausible; for example, 120 % w/v E-Z-HD barium is described as being similar in viscosity to water in one study [51], although barium solutions typically have non-Newtonian flow characteristics and viscosities well above those of water [54, 55]. The fact that both starch- and xanthan-gum thickeners are acknowledged to produce liquids with non-Newtonian flow [54, 56–62] presents a challenge when comparing the stimuli used across these studies; the measured value of viscosity (i.e., “apparent viscosity”) is very sensitive to the shear rate at which the measurement is taken. In cases where viscosity measures were reported, the literature lacked any apparent convention with respect to reporting values at specific shear rates. From the data reported, it can be noted that the non-opaque stimuli labeled as “thin” had viscosities ranging up to 12 mPa s @ 45/s [28], while the radio-opaque “thin” liquid stimuli spanned a larger viscosity range, reaching reported values as high as 351 mPa s at 25/s [29]. Non-opaque liquids described as mildly thick or nectar-thick had viscosities as high as 466 mPa s at 25/s [29] or 325 mPa s at 45/s [28], while the radio-opaque liquids in this category had viscosities up to 863 mPa s at 25/s [29]. Similarly, the stimuli labeled as moderately thick or honey-thick had viscosities reaching 1,541 mPa s at 25/s [29] for radio-opaque liquids or 785 mPa s at 45/s [28] for non-opaque stimuli. It is interesting that even among manuscripts arising from the same lab [28, 29] there is no clear convention regarding the shear rates at which viscosities are reported. Shear rate is the term used to describe the rate of deformation of non-Newtonian stimuli as the fluid layers slide over each other when the bolus is placed under stress or force. During swallowing, shear rate for a bolus may be altered by the speed of biomechanical events including tongue movement and pharyngeal shortening or constriction. Perceptual experiments suggest that a range of shear rates is likely to be operational in the mouth during oral processing and swallowing [59, 63, 64]. However, there is no clear guidance from the literature regarding the shear rates that should be used as references when reporting the apparent viscosities of food and fluid stimuli that are being studied. Such variation in reporting makes for confusion and limits generalizability across studies.

### Risk of Bias

The evaluation for risk of bias was performed according to the guidelines suggested by the Cochrane Bias Methods Group [65]. Specifically, the methods of each study were reviewed to determine whether there was potential bias in terms of participant selection, the performance of the particular study tasks by the participants, the detection or measurement of behaviors of interest, attrition or missing data, and reporting of results. As shown in Table 6, for the 36 studies reviewed, there were identified risks with respect to bias for every single study. By far, the most common risk of bias lay in the failure to report whether or not raters were blind to bolus consistency during analysis. In some cases, blinding to participant identity was reported, but given the nature of our interest in determining whether there are objective differences in swallowing or oral processing behaviors across boluses with different textures, blinding to stimulus consistency is an important consideration. It may well be that in some cases, such as videofluoroscopy, blinding to bolus consistency is less practical or feasible; however, the literature reviewed lacked acknowledgment of this issue entirely. This may reflect the fact that the primary question in many of these studies was something other than measuring differences in swallowing as a function of bolus consistency; nevertheless, in future studies where this is the purpose, blinding to bolus type would be desirable to limit bias during data analysis. Similarly, in the majority of cases, the reported data appeared to arise from analysis by a single rater with no reporting of inter- or intra-rater reliability. In some cases, measures appeared to be taken online and involved some degree of subjectivity, such that measurement validity and reliability are concerns for many of the studies reviewed. Finally, a subtle but important risk of bias must be mentioned regarding this literature to the extent that investigators selected particular stimuli to study and the reasons guiding these choices were not always reported. As described in the previous section, the stimuli covered by this literature represent a wide variety of discrete points along any sort of viscosity or material characteristic continuum. As such, caution is warranted in drawing conclusions that may be generalized to other ranges on these continua.

**Table 6:** Summary of risk of bias assessments

Author	Risk of bias?	Type of bias
Anderson et al. [39]	+	Blinding to bolus type during data processing not disclosed; no information regarding reliability of measurements
Ashida et al. [44]	+	Very small sample (*n* = 8); rater blinding to bolus type not disclosed; insufficient statistical detail reported to determine whether repeated measures were handled correctly
Bisch et al. [22]	+	Protocol incomplete for some participants; rater blinding to bolus type not disclosed
Barata et al. [48]	+	Referred sample (limited generalizability); rater blinding to bolus type not disclosed; no information regarding reliability of ratings
Bingjie et al. [24]	+	Rater blinding to bolus type not disclosed; no information regarding reliability of ratings
Butler et al. [33]	+	Rater blinding to bolus type not disclosed; no information regarding reliability of ratings
Chen et al. [51]	+	Referred sample with questionable generalizability; Rater blinding to bolus type not disclosed; no information regarding reliability of ratings
Chi-Fishman and Sonies [53]	+	Rater blinding to bolus type not disclosed; no information regarding reliability of ratings
dos Santos et al. [20]	+	Referred sample with questionable generalizability; rater blinding to bolus type not disclosed; no information regarding reliability of ratings
Funami et al. [43]	+	Very small sample (*n* = 7); single trial per bolus type; insufficient detail regarding processing of EMG and acoustic data reported; no information regarding reliability of measures
Gisel [18]	+	Rater blinding to bolus type not disclosed
Goldfield et al. [17]	+	Referred sample with questionable generalizability; rater blinding to bolus type not disclosed; no information regarding reliability of ratings
Hoebler et al. [42]	+	Rater blinding to bolus type not disclosed; no information regarding reliability of measures; insufficient statistical detail reported to determine whether repeated measures were handled correctly
Igarashi et al. [30]	+	Rater blinding to bolus type not disclosed; no information regarding reliability of rating; some data excluded due to poor quality signal
Inagaki et al. [36]	++	Measurement of sEMG from tongue surface is not validated; rater blinding to bolus type not disclosed; no information regarding reliability of ratings
Inagaki et al. [37]	++	Measurement of sEMG from tongue surface is not validated; rater blinding to bolus type not disclosed; no information regarding reliability of ratings
Inagaki et al. [38]	++	Measurement of sEMG from tongue surface is not validated; rater blinding to bolus type not disclosed; no information regarding reliability of ratings
Ishida et al. [31]	+	Rater blinding to bolus type not disclosed; no information regarding reliability of ratings; handling of missing data queried
Karkazis and Kossioni [41]	+	Rater blinding to bolus type not disclosed
Karkazis and Kossioni [47]	+	Rater blinding to bolus type not disclosed; no information regarding reliability of ratings
Karkazis [46]	+	Very small and select sample (*n* = 6); rater blinding to bolus type not disclosed; no information regarding reliability of ratings
Kim and Han [21]	+	Rheological measures not fully described
Lee et al. [52]	+	Exclusion of severe aspirators; rater blinding to bolus type not disclosed; no information regarding reliability of ratings
Lee et al. [32]	+	Subjectivity possible in verification of signal segmentation. Some signals excluded due to poor quality
Lin et al. [49]	+	Rater blinding to bolus type and time point of measures not disclosed
Linden et al. [25]	+	Very tiny sample (*n* = 3), only women; some data excluded without explanation; rater blinding to bolus type not disclosed; no information regarding reliability of ratings
Nagatomi et al. [40]	+	Subjective determination of endpoint of chewing cycle; insufficient statistical detail reported to determine whether repeated measures were handled correctly
Oommen et al. [23]	+	Exclusion of some data on the basis of subjective judgment of video quality; rater blinding to bolus type not disclosed
Reimers-Neils et al. [26]	+	Unbalanced sample (4 females, 1 male); rater blinding to bolus type not disclosed; no information regarding reliability of ratings
Ruark et al. [19]	+	Female sample only; rater blinding to bolus type not disclosed
Saitoh et al. [45]	+	Fixed order of presentation; single trial per bolus type per position condition; rater blinding to bolus type not reported; no information regarding reliability of ratings
Steele and Van Lieshout [28]	+	Very small sample (*n* = 8); some data lost due to sensor coil breakage
Steele and Van Lieshout [29]	+	Very small sample (*n* = 8); some data lost due to sensor coil breakage
Taniwaki et al. [27]	+	Details regarding segmentation method unclear; rater blinding to bolus type not disclosed; no information regarding reliability of ratings
Troche et al. [50]	+	Rater blinding to bolus type not disclosed
Youmans et al. [35]	+	Online reading of pressures with no information regarding inter-rater or intra-rater reliability. No blinding to bolus type

### Observed Trends and Levels of Evidence

Notwithstanding the caveats mentioned in the previous three sections, the identified studies do provide sufficient preliminary information to support a trend analysis regarding differences in swallowing physiology and function related to differences in stimulus consistency. Table 7 summarizes the main findings from each of the 36 reviewed studies, which are grouped according to the type of instrumentation used to measure swallowing or oral processing behavior. Videofluoroscopy and surface electromyography were used in 12 and 10 studies, respectively, thereby accounting for the bulk of the observed trends, but in total, 12 different types of instrumentation were used.

**Table 7:** Summary of study results, listed by technology, with levels of evidence rated according to the scheme of the National Health and Medical Research Council of Australia [66]

Author	Sample size	Technology	Finding	Level of evidence
Lee et al. [32]	17	Accelerometry	Accelerometry signals exhibited a more prominent, well-defined pattern as bolus viscosity increased. Nectar-thick and honey-thick apple juices were associated with longer swallow durations on average than water and thin barium	IV
Steele and Van Lieshout [28]	8	Articulography	Greater variation in tongue movement for honey-thick items and least for thin items	IV
Steele and Van Lieshout [29]	8	Articulography	Longer tongue movement durations and higher variability seen with honey-thick liquids compared to the nectar and thin	IV
Taniwaki et al. [27]	6	Auscultation/acoustics	Sounds associated with swallowing water were of longer duration and of higher intensity for water than for yogurt and konjac jelly	IV
Anderson et al. [39]	26	Camera recordings of chewing behavior	Greater muscular effort when chewing harder gum produces a greater excursive range and velocities of mandibular movement except during the occlusal phases of chewing when the harder gum slows the mandible	IV
Gisel [18]	143	Camera recordings of chewing behavior	Texture determined very strongly how long a bite of food was chewed, with solids taking longest, followed by gelatin and puree, respectively. As children became older they became more efficient at chewing a comparable bite of food, i.e. chewing time decreased for each texture	III-2
Barata et al. [48]	23	Naso-endoscopy	Thicker consistencies and solid foods were more likely to lead to residue. Thicker consistencies and solid foods were less likely to elicit laryngeal penetration/aspiration and nasal regurgitation	IV
Butler et al. [33]	22	sEMG	Viscosity (honey-thick vs. thin] did not alter swallow apnea duration in healthy adults	IV
Igarashi et al. [30]	12	sEMG	Overall trend for longer durations of sEMG and laryngeal movement with increasing thickness	IV
Inagaki et al. [36]	9	sEMG	Tougher and more adhesive foods prolonged the duration of anterior tongue, but not suprahyoid muscle activity during swallowing in normal subjects	IV
Inagaki et al. [37]	9	sEMG	Foods of thicker consistency elicited a trend toward higher integrated suprahyoid sEMG amplitude and longer sEMG durations	IV
Inagaki et al. [38]	9	sEMG	The swallowing of harder and more adhesive foods was associated with stronger integrated and cumulated anterior tongue and suprahyoid EMGs. EMG activity increased in a stepwise fashion as the concentration of the thickening agent rose from low to high	IV
Karkazis [46]	6	sEMG	Findings agree with those reported in dentate subjects and complete denture wearers: harder foods require higher chewing rates, higher EMG activity and higher relative contraction times, accompanied by shorter cycle durations	IV
Karkazis and Kossioni [41]	22	sEMG	The mean values for integrated EMG, duration of chewing cycle, the chewing rate and the relative contraction time during swallowing were significantly higher for the carrots compared to the gum. A strong inverse correlation was found between chewing rate and cycle duration. Adjustments to food consistency are made by altering chewing rate, the duration of the chewing cycle and integrated EMG activity	IV
Karkazis and Kossioni [47]	9	sEMG	In experienced denture wearers, harder foods (i.e. carrots) showed higher rates of chewing, higher masseter EMG measures of muscle force and shorter cycles than softer foods (apple)	IV
Reimers-Neils et al. [26]	5	sEMG	Thick paste stimuli elicited significantly longer “swallow duration” (from sEMG) compared to liquids and thin pastes. Multi-peaked sEMG patterns (rather than single peaked patterns) were more common with the thick pastes. Peak amplitudes for both submental and infrahyoid EMG were higher for the thick paste consistency compared to both liquids and thin pastes	IV
Ruark et al. [19]	30	sEMG	Submental and strap muscle activity were longer for cheese spread compared to water. Strap muscle activity was longer for pudding and cheese spread versus water. Amplitude was also higher for cheese spread than the other stimuli	III-2
Nagatomi et al. [40]	12	Texture profile analysis after oral processing	Changes in the mechanical properties of the bolus due to oral processing are dependent on the texture of the food. All foods appear maintain a constant level of cohesiveness across oral processing (0.5). Immediately before swallowing, all three test foods had similar factor structures based on 5 mechanical properties studied using principal component analysis	IV
Hoebler et al. [42]	12	Texture profile analysis after oral processing	The dry matter content of the food bolus influences the chewing time but is not the only variable to take into account. The size reduction of food, its de-structuring and the rate of starch hydrolysis depends on the chewing time as well as the physical characteristics of ingested food	IV
Youmans et al. [35]	96	Tongue pressure measurement	Comparisons within volume show clear trends for increasing maximum swallowing pressure and percent maximum swallowing pressure from thin to nectar to honey to puree	III-2
Chi-Fishman and Sonies [53]	31	Ultrasound	Spoon-thick liquids elicited longer durations of hyoid shadow movement than thin and nectar	IV
Bingjie et al. [24]	205	VFSS	Penetration and aspiration frequency reduce as consistency becomes thicker. Oral transit times are longer for bread than for liquid consistencies (thin and paste). Pharyngeal transit time increases from thin to paste to bread in healthy adults. Pharyngeal delay is shorter for paste and bread than for thin in healthy adults. In stroke patients who aspirate, pharyngeal delay and pharyngeal transit are longer for paste and for bread than with thin liquids. In healthy adults, there are trends towards larger hyoid and laryngeal excursion from thin to paste to bread	III-2
Bisch et al. [22]	28	VFSS	Pudding elicited significantly longer UES opening durations and significantly shorter duration of tongue base contact. Pharyngeal delay time was significantly shorter with pudding in the stroke patients. See chart in later worksheet	III-2
Chen et al. [51]	41	VFSS	Frequency of aspiration in patients studied decreased as viscosity increased	IV
dos Santos et al. [20]	32	VFSS	No dramatic trends related to texture in either group	III-2
Goldfield et al. [17]	10	VFSS	Nectar thick barium flows more slowly through the pharynx than barium intended to simulate breast milk in NICU babies	IV
Ishida et al. [31]	12	VFSS	There were no differences in forward or upward displacement of the hyoid across the 4 solids tested	IV
Lee et al. [52]	29	VFSS	Mixed consistency was less likely to be aspirated than thin and more likely to be aspirated than rice. Residue was more likely for rice and mixed than for thin. Pharyngeal delay time was longer for mix compared to rice. Penetration–aspiration was significantly worse for mix than for rice, but better than for liquid. Location of bolus at swallow onset for mixed matched that seen for liquid	IV
Lin et al. [49]	20	VFSS	Oral transit times were longer for paste consistency than for thin barium. Pre-treatment (functional electrical stimulation), hyoid displacement durations were shorter and vallecular residue was greater for paste consistency than for thin barium	IV
Linden et al. [25]	3	VFSS	On average, the bolus head was further advanced into the pharynx (past the faucial pillars) with the paste versus the thin liquid barium, in these three patients. Not clear whether they used command swallow paradigm	IV
Oommen et al. [23]	72	VFSS	Thin versus nectar-thick barium did not alter stage transition duration or laryngeal closure duration in stroke patients	III-2
Saitoh et al. [45]	15	VFSS	Chewing and initial consistency altered the relationship between food transport and swallow initiation. When liquids are chewed, or when consuming mixed consistencies, a portion of the bolus reaches the hypopharynx before swallow onset. Chewing reduces the effectiveness of the posterior tongue-palate seal, allowing oral contents to spill into the pharynx	IV
Troche et al. [50]	10	VFSS	Pudding-thick consistency was associated with significantly longer oral transit times, a greater number of tongue pumps per bolus and lower (better) PAS scores than thin barium. There were no significant differences in pharyngeal transit time	IV
Funami et al. [43]	9; 7	Multiple methods: mechanical bolus compression; sensory profiling; sEMG; acoustics	Duration of oral processing (based on suprahyoid EMG activity) for both gels was longer than for data on water, and increased with increased gel hardness. Acoustic data suggested more rapid bolus flow for the mixed gels than for the simple gels. Mixed gels were rated to have higher cohesiveness and greater ease of swallowing than simple gels. Differences in textural attributes of these gels exist even when hardness is uniform	IV
Kim and Han [21]	20	Multiple methods: salivary measures and viscosity measures after oral processing	Foods differ in viscosity pre and post oral phase, based on holding in the mouth (no chewing other than thick rice gruel). Demonstrates that viscosity becomes lower as a function of the oral phase. Stroke patients chewed more, had longer oral phases	III-2
Ashida et al. [44]	18	Multiple methods: sEMG and texture profile analysis after oral processing	Chewing time and number of chewing cycles were correlated with hardness of the stimuli (longer chewing for increased hardness). Hardness and other rheological properties of agars do not affect normalized measures of cumulative masseter muscle EMG amplitude and duration, based on analysis of the first and last chewing cycles in chewing sequences	IV

Levels of evidence: III-2: evidence from comparative studies with concurrent controls without randomized allocation (cohort studies), case–control studies, or interrupted time-series with a control group; III-3: evidence from comparative studies with historical control, two or more single-arm studies, or interrupted time-series without a parallel control group; IV: evidence from case series, either post-test or pre-test and post-test, or superseded reference standards; V: expert opinion, physiology, bench research or “first principles” studies

*sEMG* surface electromyography, *VFSS* videofluoroscopic swallowing study

The level of evidence for each main finding is shown in the far right column of Table 6, according to the scheme used by the National Health and Medical Research Council of Australia [66]. It can be noted that the selected studies fall into one of two types with respect to level of evidence. In total, 28 studies [17, 25–33, 36–53] were classified as reporting level IV evidence, that is, evidence arising from case series, post-test or pre-test and post-test studies without any comparison to controls. The remaining 8 studies [18–24, 35] were classified as level III-2 studies, reporting evidence from non-randomized cohort, case–control or interrupted time-series studies involving comparison to a control group.

Comparing results across studies, it is possible to identify patterns associated with thickened liquids or food texture modification. With respect to liquids, thicker liquids were reported to increase the duration of swallowing events compared to thin liquids in accelerometry [32], electromagnetic articulography [29], ultrasound [53] and surface electromyography signals [19, 26, 30], and also on videofluoroscopy for pharyngeal transit time measures [17, 24]. In patients with stroke-related dysphagia, longer upper esophageal sphincter opening durations [22] were also reported for paste consistency stimuli than with thin liquids, while longer oral transit times were observed with the paste consistency in patients with Parkinson’s disease [50] and those who had received radiation therapy for nasopharyngeal carcinoma [49]. Electromyographic measures of oral processing duration were longer for agar gels compared to water data [43]. Findings regarding the influence of liquid consistency on pharyngeal delay times in stroke patients were equivocal, with one study reporting longer delays with a pudding-thick consistency [24] and a second study reporting the opposite trend [22]. Two further reports found results that conflicted with the generally observed trend of longer duration events being seen with increasing viscosity. One study reported that the sounds associated with swallowing water were longer than those seen with either yogurt or konjac jelly [27]. Hyoid movement durations were also described to be shorter with paste consistency compared to thin barium following radiation treatment for nasopharyngeal carcinoma [49]. Two studies describe measures that did not change as a function of liquid consistency: swallow apnea duration was reported to remain unaffected by bolus consistency in healthy adults [33] while measures of swallow response time (also known as stage transition duration) and laryngeal vestibule closure duration did not differ for a thin to nectar-thick liquid barium comparison in stroke patients [23].

In addition to observations regarding physiological timing measures, other reported measures support the impression that thicker and harder items require greater effort in oral processing and swallowing. Measures that contribute to this observation include more prominent and well-defined accelerometry signal peaks [32], greater variability in tongue movement patterns [28, 29], higher surface electromyography amplitudes [19, 36, 41, 47], higher velocities of jaw movement [39], greater variability in surface electromyography patterns [26], and increased amplitudes of tongue-palate pressure [35]. Several studies concur that boluses with increased hardness elicit timing differences in chewing, involving faster rates, longer cycle durations, and a greater number of cycles [18, 41–44, 46, 47]. Findings were mixed with respect to the influence of bolus consistency on the magnitude of hyoid and laryngeal movements. One large study reported larger hyoid and laryngeal excursion for paste consistency and bread boluses compared to thin liquids [24], while a smaller study in healthy adults failed to find differences across different solid boluses [31].

**Fig. 2:**
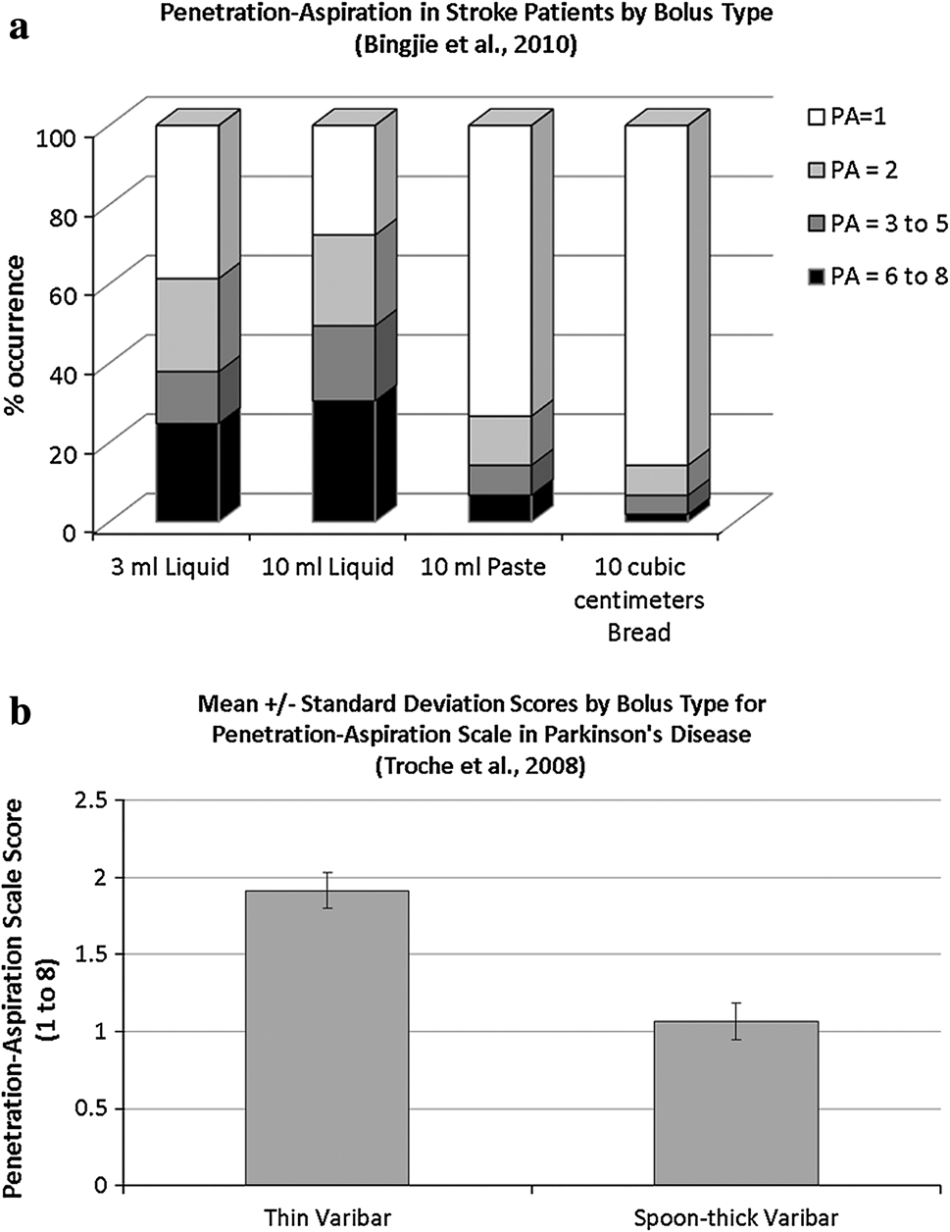
**a** Prevalence of penetration–aspiration by liquid bolus consistency, as reported in a study of stroke patients by Bingjie et al. [24]. Penetration–aspiration scale scores of 1 and 2 are considered normal; scores of 3–5 indicate penetration of the laryngeal vestibule, while scores of 6–8 indicate aspiration of material below the true vocal folds. **b** Differences in the severity of penetration–aspiration as a function of liquid bolus consistency, as reported in a study of patients with Parkinson’s disease by Troche et al. [50]

With respect to functional swallowing measures, an important question is to determine whether penetration–aspiration of material into the airway is effectively reduced by altering bolus consistency? Several of the videofluoroscopic studies concur on this question, as illustrated in Fig. 2a, b. Bingjie and colleagues reported that the frequency of penetration–aspiration in stroke patients decreased as liquid viscosity increased [24]. This trend was also seen in the studies by Chen et al. [51], Barata et al. [48], Troche et al. [50] and by Lee et al. [52], who further described that aspiration was worst for thin liquids, better with a mixed consistency involving rice in liquid barium, and best for rice served without combining it with liquid. However, a cautionary note is also warranted on the basis of the selected studies, in that greater vallecular residue was observed with paste consistency barium than with thin liquid barium [48, 49] and with a plain rice bolus compared to a rice and barium mixed consistency [52]. Troche and colleagues [50] also observed that patients with Parkinson’s disease used a greater number of tongue pumps to successfully swallow a pudding-thick consistency, than for a thin liquid bolus, suggesting that clearance was worse with the thicker consistency. A recent report by Hind and colleagues [67], also reports a trend toward greater pharyngeal residues for barium stimuli with increasing viscosity.

An interesting study exploring swallowing with liquid barium, mixed consistency and a solid food (corned beef) demonstrated that for mixed consistencies and the solid food, the location of the bolus at swallow onset was lower in the hypopharynx than with liquids [45]. However, in a clever twist in their experimental design, these authors also asked participants to engage in chewing with the liquid barium stimulus and showed that this led to accumulation of the liquid bolus in the vallecular space, as seen with the mixed and solid consistencies.

With respect to solid foods, the literature search identified several studies in which rheological or texture profile analysis methods were used to measure the characteristics of the food bolus were at the end of oral processing, when the bolus was considered to be ready for swallowing [21, 40, 42–44]. These articles suggest that the property of cohesiveness remains stable during chewing and oral processing while other mechanical properties change [40] and are influenced by dry matter content [42], the degree to which salivary enzymes are absorbed by the bolus and contribute to starch hydrolysis [42], and the composition of the bolus with respect to the use of single gelling agents or complex gel combinations [43, 44]. In the food oral processing literature, the construct of cohesiveness is defined as a mechanical textural attribute relating to the degree to which a substance can be deformed before it breaks. The standard method for measuring cohesiveness during sensory panel testing involves placing a sample between the molar teeth, compressing the sample, and evaluating the degree of deformation before rupture [68–70]. Adjectives that are listed as descriptors of cohesiveness include: fracturable, crumbly, crunchy, brittle, crispy, crusty, chewy, tender, tough, short, mealy, pasty, and gummy. The texture reference scale developed by Munoz [71] is recommended in ISO guidelines for sensory ratings of cohesiveness, but it is acknowledged that no suitable set of reference products has been developed for this attribute.

## Discussion

### Evidence Supporting or Refuting Thickening of Liquids

Collectively, the selected studies clearly show a reduction in the risk of penetration–aspiration with liquids, as they progress from the thin to the very thick end of the viscosity continuum. This finding is limited, by definition, to studies in which objective measures of penetration–aspiration were available, which for the purposes of the present review meant studies involving barium swallowed under videofluoroscopy. Evidence regarding penetration–aspiration was also limited to studies involving adult participants with dysphagia.

However, an important cautionary note arises from this review given the convergence of evidence across several studies, showing a heightened risk of post-swallow residue in the pharynx for liquids with higher viscosities. This points to an important clinical challenge in terms of identifying suitable and safe consistencies for patients with dysphagia; namely, that of identifying liquids that are thick enough to be swallowed safely (without penetration–aspiration) while avoiding the pitfall of post-swallow residue.

As a post-script on this particular question, an additional source of data was brought to the attention of the authors after completion of the qualitative synthesis of the selected articles. This as-yet unpublished doctoral dissertation [72] involved rigorous videofluoroscopic exploration of swallowing with thin and nectar-thick Varibar™ barium by infants aged 3 weeks to 3 months, referred for evaluation of swallowing secondary to respiratory compromise. This dissertation is particularly noteworthy given the relative dearth of information regarding pediatric feeding and swallowing uncovered in our search process [17–19]. The Gosa study [72] reports several findings that concur with the observations gleaned from our qualitative synthesis, including prolongations of oral transit time and a reduction in penetration–aspiration with the nectar-thick stimulus compared to the thin liquid barium. Additionally, residue was reported to be present for 80 % of the nectar-thick swallows compared to only 44 % of thin liquid swallows. An additional gap to highlight with respect to the lack of identified studies in either the healthy or dysphagic infant population is the challenge of matching assessment stimuli to the rheological properties of breast milk or infant formula. This is a question of emerging interest in the dysphagia literature [9, 73] and definitely an area where additional research is needed.

### Evidence Regarding the Number and Definitions of Levels of Liquid Thickening

Although this systematic review finds a convergence of evidence showing that thicker liquids are less likely to be aspirated, and more likely to cause post-swallow residue, the available data are insufficient to suggest particular viscosity values or other quantitative measures of material properties along the continuum from thin to extremely thick liquids that represent boundaries of clinical importance [74]. Historically, clinical guidelines regarding the use of thickened liquids have proposed quantitative boundaries that arise either from clinical consensus, or represent an educated guess. For example, the National Dysphagia Diet in the United States proposes 4 levels of liquid viscosity, labeled “thin”, “nectar-thick”, “honey-thick” and “spoon-thick” and corresponding to apparent viscosity ranges of 1–50, 51–350, 351–1,750, and ≥1,751 mPa s, measured at a shear rate of 50/s [10]. The Japanese guideline provides a larger number of categories with viscosity ranges of 1–50, 51–150, 151–300, 301–500, and >500 mPa s, again measured at a shear rate of 50/s [14, 15]. In the current review, we found no specific evidence to support or refute the specific numeric categorical viscosity boundaries suggested in these or other guidelines. We found no evidence to suggest that there are transitions of clinical relevance occurring at the boundaries between categories in these guidelines. Furthermore, the available evidence to date does not help us to ascertain how large a viscosity difference needs to be, in order to have a beneficial and measurable effect with respect to reducing penetration–aspiration, nor at what point the risk of residue accumulation becomes a real concern. Similarly, the available evidence does not provide clear evidence to suggest how many incremental levels of increasing viscosity might be meaningful in the clinical context. The available data also lack evidence regarding the important possibility that properties of a liquid bolus other than apparent viscosity, such as density, yield stress, cohesiveness, or slipperiness (to list a few) might influence swallowing physiology and function. On this latter point, we are aware of a recent publication describing differences in the rates of occurrence of penetration–aspiration for liquids, depending on the type of thickener used (starch vs xanthan-gum), albeit thickened to different degrees [75]. A recent conference abstract also reports differences in residue accumulation for liquids thickened with corn-starch versus xanthan-gum thickeners, and attributes these differences to subjectively judged cohesive properties of the bolus [76]. Similarly, several articles reviewed in this study revealed that different thickening agents produced products that had different rheological or material property profiles, as suggested in prior studies [60], and were shown to require different degrees of oral processing and suggested to have different rates of flow [43, 44]. Thus, it is naïve and not appropriate to assume that liquids thickened to similar viscosities using different agents will behave similarly in the oropharynx. The possibility that properties other than viscosity may have clinical relevance is both intriguing and important, and poses a challenge to the scientific community to develop rigorous studies that characterize such properties according to validated methods, in order to explore such phenomena.

Given the recognition that particular numeric viscosity boundaries for levels of liquid thickening are neither empirically supported nor empirically refuted, we conclude that the most appropriate clinical course of action with respect to identifying the optimal consistency of liquids for a patient who aspirates thin liquids is to increase viscosity in relatively small increments until swallowing safety is demonstrated. How large these increments might need to be can perhaps be informed by evidence from the sensory literature, based on the assumption that changes in behavior result from perceived differences in bolus consistency. The literature shows that the scaling of oral viscosity perception does not grow in a linear manner, but rather in an exponential fashion [77, 78]. Human ability to discriminate viscosity is proportional to the viscosity of the sample itself, as described by Weber’s law [79]. Recently, Withers and colleagues [80] manipulated the cream/fat content, and viscosity of skimmed milk using 0.1 % w/v increments of a starch-based thickener to explored the thresholds of just-noticeable differences (JND) in perceived thickness by healthy adults. They found no age differences in viscosity discrimination for liquids with apparent viscosities between 45 and 135 mPa s at 44/s, and reported that the average JND was between 0.26 and 0.32 % w/v in terms of thickener concentration, which equated to approximately a 1.8-fold increase in apparent viscosity (i.e., from 45 to 83 mPa s). Another recently published study explored just-noticeable differences using 0.1 % w/v increments in the concentration of a xanthan-gum thickener for sweetened cordial stimuli between 190 and 380 mPa s at 50/s [56]. In this case, the JNDs were found to be narrower, namely 0.38-fold for thickener concentration, equating to a 0.67-fold increase in viscosity [56]. Differences in the nature of the liquids studied (i.e., dairy products vs cordials), the viscosity ranges probed (i.e., 45–135 vs. 190–390 mPa s) and the testing methodologies used (i.e., two-stimulus forced choice comparisons versus three-stimulus triangle test paradigms) may have contributed to the observed differences in the resolution of perceivably different viscosities across these two studies. The authors of the second study [56] extrapolated from their findings to suggest that an array of liquids with apparent viscosities of 5, 8, 13, 22, 36, 60, 100, 170, 280, 470, 790, 1320, and 2200 mPa s at 50/s might provide a starting point for evaluating the influence of perceivably different viscosities on swallowing. However, it should also be noted that all of the viscosity discrimination studies cited [56, 77, 78, 80] were conducted using healthy volunteers with intact sensory systems. Alterations to oral or pharyngeal sensation, such as may be seen in individuals with dysphagia secondary to stroke, may alter perceptual viscosity discrimination abilities compared to healthy individuals. It should also be noted that the perceptual thresholds for noticeable viscosity differences arising from both of these studies are smaller than the categorical boundaries suggested by current clinical guidelines. Certainly, the results of our qualitative synthesis point to a significant gap both in literature and knowledge regarding the impact of small increments of viscosity on swallowing, and illustrate the need for new studies, which explore both the physiological and functional consequences of thickening in both narrow and larger increments.

As a final comment in this section on thickened liquids, some attention is required on the issue of viscosity measurement and its dependence on shear rate, which is a quantified measure of the speed of flow. The addition of either starch or xanthan-gum thickeners to liquids will result in non-Newtonian characteristics [56–62], meaning that the apparent (measured) viscosity is strongly dependent on shear rate. This is also true of most, but not all contrast media used for swallowing evaluation [54, 55]. Thus, if a measure of viscosity is reported, the shear rate used in the measurement is critical to understanding the measurement and to enabling comparison across studies. Although it has been common to report apparent viscosity at a shear rate of 50 reciprocal seconds (i.e., 50/s) [10, 14, 56], none of the studies identified for inclusion in this systematic review followed this convention. Notably, available information regarding the viscosity of Varibar™ (a line of commercially available barium products for swallowing evaluation used frequently in the United States) is quoted at a shear rate of 30/s. The actual shear rates involved in oral processing and swallowing depend on the rate and degree of pressures applied as well as the material properties of the fluids, and as such, can vary widely. The oral preparatory phase probably involves low shear rates (particularly for thicker fluids) and the perception of thickness has been shown to be best-related to objective measurements of viscosity taken at 10/s [64]. The pharyngeal and esophageal stages of swallowing are thought to involve much more rapid flow, with computer simulations suggesting shear rates in the order of 400/s for water [81].

Until such time as new research is available to describe the shear rates that are actually operating during swallowing [82], both in healthy and impaired contexts, it is paramount that apparent viscosities of thickened liquids intended both for assessment and therapeutic clinical purposes be reported across a range of shear rates. As a starting point, we recommend that shear rates of 1, 10 [64], 30, 50, and 100 reciprocal seconds would provide a reasonable basis for comparison. We particularly encourage consideration of liquid flow behaviors at low shear rates due to the likelihood that motoric deficits in dysphagia may impact a person’s ability to generate the shear forces and physiological behaviors that are typical of healthy swallowing.

### Evidence Supporting or Refuting Texture Modification of Foods

If the literature on thickened liquids is sparse, this is even more apparent when reviewing the literature regarding texture-modified foods and swallowing. As illustrated in Table 5, the identified literature discussed only a small number and variety of solid foods. With the exception of longer duration and higher amplitude masseter surface electromyography signals when ingesting solid foods with increasing thickness or hardness [19, 30, 36–38, 43, 44, 46], the findings of the identified studies do not clearly point to measurable differences in either oral processing or swallowing parameters across the particular solid foods tested. We did not, for example, find literature that specifically explored the particle size of solid foods after a specific timeframe of chewing by people with partial or missing dentition or with reduced chewing strength. Penman and Thomson suggest that particles of 1.5 cm^2^ constitute a choking hazard for people with dysphagia [83], but the studies that we found describing the characteristics of solid foods after oral processing focused more on textural profiling than on particle size. Although this information may exist in the dental or food oral processing literature, it was not found given the specified search strategy, and, as previously acknowledged, the term “chok*” was not included in our search. Data regarding solid food particle size after oral processing under both normal and abnormal dental conditions would be interesting to consider alongside autopsy results suggesting that individuals with partial or missing dentition are more prone to choking on food [84]. A recent report by the Japanese Food Safety Commission [85], concludes that food texture (surface smoothness, elasticity, hardness), size, and shape are all relevant with respect to choking risk. In their investigations, sticky rice cakes were found to be the leading cause of choking accidents, but jelly cups were also mentioned as a not infrequent cause of choking. The report highlights that the risk of choking on a particular food item needs to be understood both in terms of the textural properties of the bolus and of the physiological behaviors commonly used during ingestion of that item. Thus, the jelly cups, for which they describe a common behavior of tilting the head backwards to suck the jelly out of the cup, are not without risk.

The review revealed common use in the food oral processing literature of accepted terminology to describe the textural attributes of solid foods as laid out in ISO guidelines [68, 69]. The construct of cohesiveness, mentioned earlier, is one example of such terminology. One term, which was encountered in the food oral processing literature, but which remains poorly understood, is “ease of swallowing” [86]. This appears to be an attribute that is commonly captured in sensory profiling of food textures; however, whether and how this attribute maps to objective, quantifiable measures of bolus flow or physiology remains unclear.

An interesting question arising from this review is whether adhesive paste consistency stimuli such as cheese spread or peanut butter should be considered to be semi-solid foods or extremely thick liquids? These items can be compressed and spread across the palate with the tongue, and do not fracture; as such, in a physiological sense, they behave quite differently and involve different oral processing behaviors from foods that require mastication [87]. On the other hand, they do not flow either under gravity, or under the typical pressures applied by the tongue, and require handling by the tongue for transport through the oral cavity. In this respect, they are quite different from liquids. It is recommended that future investigations with respect to differences in oral processing and swallowing of solid foods and thickened liquids make a clear distinction based on the physiological processes that are required for transport (i.e., mastication, oral containment, tongue-sweeping, and propulsion or simple tongue compression), rather than using texture descriptors derived based on physical properties alone [88]. Furthermore, it may be important to note that a given stimulus may behave more like a liquid for one person and a semi-solid for another person, based on the person’s ability to generate forces or movement with their tongue. As such, physiological definitions of texture may have different boundaries for different consumer groups. Considerations of temperature inside the mouth and the slipperiness of the oral surfaces given differences in the levels of saliva across the duration of oral processing are undoubtedly also relevant to developing a texture classification system for the dysphagia population, which is founded on a physiological framework.

The paucity of studies captured in our search describing oral processing or swallowing of texture-modified foods comes as both a disappointment and a surprise. On reflection, we believe that the rules of our search strategy may have overly limited the search results, given mandatory inclusion of the MeSH terms “Swallowing” or “Deglutition” or “Dysphagia”, even when the supplementary search for articles was performed with the additional MeSH heading term of “food texture”. We are aware that there is an entire field of scholarship known as “food oral processing”, with its own journals and conferences. Although our search strategy employed search engines intended to tap the engineering and non-medical domains, it may well be that key words related to swallowing and dysphagia are not commonly used in publications within this subspecialty, leading our search to capture only a limited number of articles from this domain. Certainly, a direction for future research will be to explore this literature in greater detail for relevant evidence regarding differences in oral processing behaviors for foods with different textural characteristics.

From a clinical perspective, the lack of guidance regarding the classification, labeling, and preparation of texture-modified foods for people with dysphagia is a concern. It is not uncommon for coroner’s inquiries into fatal choking episodes in people at risk for dysphagia to conclude that food of an inappropriate consistency was ingested [84, 89–91]. On the basis of the current review, we are obliged to point out that the best available evidence regarding the selection of an optimal food consistency for a person with dysphagia comes from the careful exploration of tolerance for different foods in a comprehensive clinical swallowing assessment. This systematic review found a lack of research evidence providing support for the selection or avoidance of specific consistencies. Our review points to an urgent need to generate empirical evidence to describe different classes of chewable food, so that the corresponding expected differences in oral processing and swallowing behavior can be defined. Additionally, the development of valid methods for observing, describing, and measuring oral stage behaviors during assessment tasks that probe a variety of different solid foods would be a valuable addition to current subjective clinical methods. Collaboration with the research field food oral processing is strongly advised as a first step in developing such methods.

## Conclusions

At the outset of this review, we identified several main questions for our investigations regarding the impact of liquid consistency and food texture on swallowing. Our first question was to determine whether evidence supports or refutes the practices of thickening liquids and modifying food textures in the context of the clinical management of dysphagia. We conclude that evidence shows a benefit associated with thickening liquids in terms of reducing penetration and aspiration, but that this benefit brings with it a risk of post-swallow residue in the pharynx with thicker consistencies. We were unable to find evidence to delineate particular boundaries in measured viscosity that may predict these clinical outcomes. We found very little evidence to guide practice with respect to different degrees of modifying solid foods for patients with dysphagia. The literature strongly suggests that there are several relevant properties of food texture for swallowing, including cohesiveness, hardness, and slipperiness.

With respect to objective measures that might be used to guide the classification of thickened liquids and texture-modified foods, our review identified an absence of convention, particularly in terms of the shear rates that are used for reporting apparent viscosity. Exceptionally limited information was available for objective measurement of texture-modified foods. Collaboration with experts in the sensory aspects of food oral processing emerges as an important direction for future research in this respect. The adoption of sensory terms and scaling methods that have become standard in the food oral processing world to capture the characteristics of foods used in dysphagia management would be a very worthwhile pursuit both for research and clinical food production.

This systematic review has identified some major gaps in our understanding of the impact of liquid consistency and food texture on swallowing physiology, both in healthy and disordered populations. Looking to the future, we conclude that classifications of these properties should take into consideration the physiological behaviors that are observed when ingesting different stimuli. Potential delineations with clinical utility include differentiating liquids into those that flow easily in the context of minimally applied tongue pressures in the mouth versus those that require more active tongue movement to initiate flow. The behavior of a bolus in the context of bolus containment, active tongue movement, or chewing (i.e., spreading vs flow vs fracture) may be another useful way of capturing clinically relevant properties of food texture for swallowing, and also appears to be relevant in terms of choking risk. These speculations raise the intriguing possibility that different boundaries of bolus texture and flow may be needed for different subpopulations within the larger clinical consumer group of people with dysphagia, depending on their physiological capabilities. Finally, this manuscript reminds us that the dysphagia field is still in relative infancy. Given the prevalent use of texture-modified foods and thickened liquids in the treatment of dysphagia, it is timely that gaps in these areas are identified and provide strong grounds for clinically relevant research to guide best practice.
